# Perceived Value Influencing the Household Waste Sorting Behaviors in Rural China

**DOI:** 10.3390/ijerph17176093

**Published:** 2020-08-21

**Authors:** Ying Ma, Mansoor Ahmed Koondhar, Shengke Liu, Huiling Wang, Rong Kong

**Affiliations:** 1 School of Economics and Management, Northwest A&F University, No. 3 Taicheng Road, Yangling 712100, China; maying@xsyu.edu.cn (Y.M.); 2018071024@nwafu.edu.cn (M.A.K.); liushengke@nwafu.edu.cn (S.L.); huilingwang@nwafu.edu.cn (H.W.); 2 School of Economics and Management, Xi’an Shiyou University, No. 18 Dianzi Road, Xi’an 710065, China

**Keywords:** waste sorting behavior, perceived value, perceived benefit, perceived cost, utility

## Abstract

Waste sorting is the cardinal measurement to solve the problem of low efficiency of rural environmental governance and to alleviate environmental pollution by reduction, recycling, and harmlessness in rural areas. However, non-excludable and non-rival features of public goods easily cause a wide free-rider problem, which results in a low frequency of participation in the waste sorting of rural people. Based on the theory of the utility maximization of the rational economic man, this paper investigates survey data of 688 farm households in three cities and three counties of Shaanxi Province to explore the effect of the perceived value on the household waste classification behavior based on cost-benefit analysis. The results show that perceived benefit and perceived cost are important perceived value factors affecting farmers’ participation in waste sorting. Specifically, the spiritual benefit of the perceived benefit has a significantly positive impact on classification behavior, while the time cost, physical cost, and material cost of the perceived cost have a negative impact on waste classification behavior. Further study of the heterogeneity of income impact shows that time cost only has a significant impact on the high-income group of farmers’ classification behavior, while spiritual benefit and learning cost only affect the low-income group of farmers’ waste classification behavior. Material cost has different influence directions on high- and low-income groups. In view of the aforementioned findings, this study highlights corresponding policy implications from the perspective of perceived benefit and perceived cost.

## 1. Introduction

In recent years, living conditions have greatly improved in rural China. However, the problem of accumulation of dirt and debris in some areas remains because garbage is not effectively processed. According to statistics, the rural population is 552 million, accounting for more than 39.4% of the total population at the end of 2019 (Published by the China National Bureau of Statistics at http://www.stats.gov.cn). The amount of garbage produced can reach 0.86 kg per person per day (Published by China Environmental Protection at http://www.hbzhan.com), and it can be estimated that the annual domestic waste production in rural areas was about 173 million tons in 2019 (resident population is 205 million). The data show that China produces nearly 1 billion tons of waste every year, including 400 million tons of domestic waste, 500 million tons of construction waste, and 10 million tons of kitchen waste. The amount of waste is growing by 8% to 10% every year in China (Published by the China Association of urban environmental sanitation at http://cnues.com).

Differently from urban waste management, rural areas are facing the following dilemmas. Firstly, compared with urban waste, the amount of rural waste is huge, and types of waste are varied. There are two main categories of rural waste in China, which are rural domestic waste and agricultural production waste. Specifically, rural domestic waste is mainly produced by farmers’ family life, including fruit peel, vegetable leaves, bones, leftovers and other kitchen waste, coal cinder, wastepaper, fiber materials, fabric, etc. Agricultural production waste is produced in the process of agricultural production, including crop straw, organic pesticides and herbicides dissolved gas, agricultural film and other plastic products, livestock and poultry breeding waste, etc. In addition, there are different methods for sorting in rural China. The methods comprise two types (recyclable and non-recyclable), three types (decomposed waste, toxic waste, and other waste), and four types (kitchen waste, recyclable waste, toxic waste, and other waste). In urban areas, only four types (kitchen waste, recyclable waste, toxic waste, and other waste) are applied. Secondly, influenced by urbanization, young rural people work as migrant workers outside their hometown, and older people and children are left behind in the rural areas. This reflects a ‘village-hollowing’ phenomenon in rural China, which leads to a large amount of garbage not collected or transported in many rural areas [1,2]. Thirdly, rural people with long-term bad waste disposal habits, including careless dumping, incineration, or landfill, aggravate environmental pollution, such as land, water, or air, and even threaten the physical and mental health of rural people [3,4]. Lastly, the low education level of the rural people makes it difficult for them to accept the new recycling facilities such as compost houses and intelligent recycling equipment, which leads to a low level of waste re-utilization in rural areas [5,6]. To solve the problems of garbage disposal in rural areas, in 2017, the Ministry of Housing and Construction of China selected 100 pilot counties to launch rural waste classification and resource utilization activities. However, the current situation is still not satisfying, and the rate of solid waste sorting is quite low [7].

Numerous studies have proved that people’s waste sorting behaviors (WSB) are affected by government instruments or organizational support of external factors [8,9], as well as personal psychological factors of internal factors [10,11]. Through regulation or policy, the government and organizations establish a waste disposal policy system limiting rural people’s free-riding behaviors caused by the positive externalities of waste treatment [12,13]. However, environment-related policies have great uncertainty, and policy outcomes may not be demonstrated immediately, which makes individuals’ policy choices change on the perceived payoffs [8,10]. Hence, more and more scholars have begun to study psychological factors. Farmers are the main participants and the direct beneficiaries of waste sorting activities. Whether the waste sorting policy succeeds largely depends on farmers’ attitude to participate in waste sorting activities [14]. As rational economic men who are ‘thin-minded,’ farmers’ decision-making is based on maximizing the utility of the trade-off between the benefits and costs of waste classification [14,15]. Perceived value is a typical psychological factor affecting households’ WSB. The perceived benefit of waste classification significantly affects the utility maximization, and then affects individuals’ WSB [14,15]. Simultaneously, the cost payment of waste sorting will decrease individuals’ utility, which then affects their enthusiasm for participating in waste sorting [16,17]. Therefore, to exploring the main psychological factor affecting the WSB of rural people, it is necessary to investigate effects of perceived value on the WSB from the perspective of benefit and cost analysis.

Although studies have investigated the perceived value influencing the WSB [8,18], the results and scopes of these studies are limited. Firstly, some scholars have paid attention to the impact of value perception on the WSB. However, it still lacks the systematic construction and measurement of perceived value indicators from the perspective of benefit and cost. Secondly, few studies have incorporated perceived cost and benefit into a framework to deeply analyze the key perceived value factors affecting people’s WSB, especially rural people. Thirdly, few scholars have focused on the analysis of the perceived value effects on the WSB at different income levels.

Based on the above analysis, perceived benefit and perceived cost of the perceived value can affect the household WSB. Besides, the household’s decision on the WSB is affected by the income level on utility theory. Therefore, there are different factors of perceived benefit and perceived cost affecting the WSB at different income levels. Hence, the purpose of the study is to explore the effect of perceived benefit and perceived cost of perceived value on household WSB and the heterogeneity effect of perceived value on the WSB at different income levels by employing ordered logit regression and using 688 data in the rural Shaanxi province, China. In addition, the study offers targeted policy proposals for improving the WSB in rural China in perspective of cost and benefit.

## 2. Literature Review and Hypothesis Development

### 2.1. Analysis of the Relationship between the Perceived Values and WSB by Utility Theory

Perceived value is an individual’s subjective perception of the process or result of a certain product or service, including the comparison and trade-off between the perceived gains and perceived losses [13,14,19]. The positive externality of environmental policy causes farmers to enjoy the environmental fruit by free riding instead of participating in environmental protection activities such as waste management [20,21]. As rational individuals and under certain budget constraints, farmers tend to decide to participate in waste separation based on the maximization of individual utility, which is measured by the value of costs and benefits of their participation in waste sorting [14,22]. Accordingly, this paper presents a literature review and hypothesis development of the perceived value effect on farmers’ WSB from the perspective of cost-benefit analysis, which is built on the theoretical assumptions of a limited rational economic man and the theory of maximizing individual utility function.

Waste sorting could produce a series of welfare effects, especially environmental and social effects, which will greatly improve individuals’ benefit perception [23]. However, all farmers share these welfare effects whether they participate or not. Therefore, farmers tend to enjoy the environmental and social ‘welfare’ of waste classification without paying the cost. Viscusi et al. believe that selective incentive strategies could encourage more people to participate in related environmental protection activities [24]. On the one hand, rural people’s behaviors are more easily encouraged by material incentive strategies [13]. On the other hand, the incentive strategy should consider a series of effective non-material incentives based on individuals’ morality and concern for others [25,26]. Hence, perceived benefits consist of material benefit and non-material benefit in this paper.

In the process of waste classification, farmers’ waste classification behaviors are affected not only by one’s benefit perception but also by cost perception [27,28]. Because of the non-exclusive feature of public goods, individuals rely more on the government, which provides public goods through taxation or fiscal expenditure instead of providing by oneself [29]. In fact, the supply of public goods in urban areas depends on the government, market, and community [30]. However, the supply of public goods in rural areas mainly depends on the government and the administrative agencies at the village and town levels [2,3]. In rural areas, the governments at the provincial and municipal levels decide the supply modes, quantity, and quality of public goods as well as services policy. The governments at the county or town level are responsible for the implementation of public goods and services. This management model squeezed out the farmers’ right to demand public goods and led to the farmers’ WSB highly depending on the government and village management organizations [3,7]. In addition, the quantity and quality of public goods or services are generally unevenly distributed between rural areas and urban areas because of the government supply difference, which means farmers have to bear material costs of public goods supply. Meanwhile, rural people will experience non-material costs, including time, energy, or study, when they participate in waste sorting. Therefore, this paper intends to measure the perceived cost of farmers participating in waste classification from the perspective of material cost and non-material cost [15,28].

According to the above analysis and the relevant literature [13], the utility model of farmers’ participation in waste classification is set as follows:

(1) $$∆U=U_{B}W_{i},S_{j}-{\;U}_{c}M_{i},F_{j}$$

In the function, U***_B_***(W*_i_*,S*_j_*) is the farmer’s perceived benefits, and U*c*(M*_i_*,F*_j_*) is the farmer’s perceived cost. Specifically, W*_i_* is the material benefits of farmers’ participation in waste classification, and S*_j_* is the non-material benefits, such as perceived environmental value of waste classification. M*_i_* is the material cost of waste sorting, and F*_j_* is the non-material cost. When ΔU > 0, that is U***_B_***(W*_i_*,S*_j_*) > U*c*(M*_i_*,F*_j_*), farmers will attend to waste classification; Otherwise, they will not participate. The perceived benefits and perceived costs include material benefits and material costs, as well as non-monetized spiritual benefits and non-material costs. Hence, based on the theoretical analysis, we put forward hypotheses from two perspectives: The perceived benefit of positive influencing factors and the perceived cost of negative influencing factors.

### 2.2. Hypothesis Development

#### 2.2.1. Impact of Perceived Benefit on Waste Classification Behavior

Perceived benefit refers to the individual’s gains in waste classification, which is a subjective perception [14]. Specifically, the perceived benefit includes spiritual benefits in terms of environmental, physical, and mental health, reputation, and other non-material benefits; and material benefits such as gifts or subsidies provided by the local government to encourage the farmers’ WSB [13,31]. Studies have shown that the higher the individual’s recognition of the perceived value of waste classification, the higher the frequency of his participation in the classification [32]. Specifically, individual recognition of egoistic benefit, social-altruistic benefit, and environmental benefit has a significantly positive impact on waste classification behaviors [33,34]. Meanwhile, as rural areas involve acquaintance societies, the reputation appeal of individuals or families will significantly affect the willingness and behavior of waste disposal [7]. In addition to the spiritual benefits, some scholars have found that the higher the subsidies for waste recycling provided by the government, the stronger the willingness to participate in waste classification, and the material benefit of waste sold has a significantly positive impact on WSB [26,35]. Hence, the higher the participants’ perceived benefits of waste classification, the more positive the attitude of classification, which could encourage them to actively participate in waste classification [19]. Thus, we hypothesize the following:

**Hypothesis** **H1a.** *Spiritual benefit has a significantly positive impact on the WSB of households.*

**Hypothesis** **H1b.** *Material benefit has a significantly positive impact on the WSB of households.*

#### 2.2.2. Impact of Perceived Cost on Waste Classification Behaviors

Perceived cost refers to individuals’ expenditure or losses in the process of waste classification [1,13], which includes the time, energy, study, and other non-material costs of waste classification, as well as the material costs in terms of the expenditure of money or material objects when they participate in waste disposal [28,36]. It was found that individuals’ perceived cost of waste classification affects their recognition of waste sorting, thus affecting their WSB [7,36,37]. Compared with the general waste treatment, waste classification is relatively complex, which will cause energy drain and reduce people’s enthusiasm for waste classification. Besides, the education level of farmers is generally low, thus developing waste classification knowledge and using recyclable equipment or intelligent garbage recovery devices will increase the farmers’ learning cost, and the higher learning cost will hinder participation in waste sorting. Studies have shown that individual classification preferences are highly related to time consumption [37,38]. Therefore, there is a significant statistical relationship between individual classification preferences and the time consumption of waste disposal. In addition to the non-material costs, when individuals spend waste treatment fees or pay a certain amount of waste treatment facility fees for waste classification, their willingness and behavior to participate in waste classification are significantly decreased [39,40]. Given the results, the following hypotheses can be suggested.

**Hypothesis** **H2a.** *Time cost negatively affects household waste classification behaviors.*

**Hypothesis** **H2b.** *Learning cost has a significantly negative impact on household waste classification behaviors.*

**Hypothesis** **H2c.** *Physical cost negatively affects household waste classification behaviors.*

**Hypothesis** **H2d.** *Material cost has a significantly negative impact on household waste classification behaviors.*

## 3. Data

**Figure 1:**
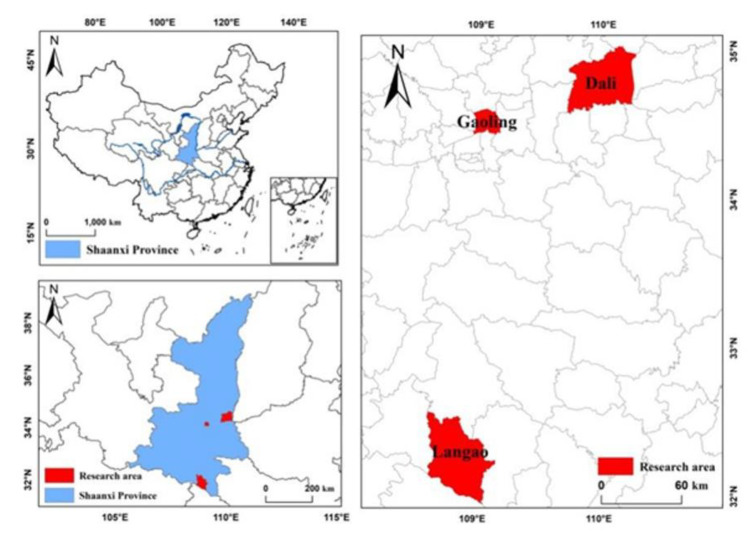
Map of the data source.

The Ministry of Housing and Construction of China chose 100 pilot counties to implement waste sorting in 2017. Four of these counties were in Shaanxi. Shaanxi is an important western agricultural area, which has a population of more than 20 million, approximately 46% of whom live in rural areas. Hence the sample of rural people of Shaanxi is representative of western China. A large-scale survey was carried out in the Shaanxi Province over the period 14–28 April 2018 (Figure 1). Considering the different rural environments of the pilot of the Baota District of Yan’an, we chose three pilot counties: Gaoling District of Xi’an, Dali County of Weinan, and Langao County of Ankang. In total, 10 administrative villages from south to central Shaanxi were selected, and 2–3 sample village (nature village) were selected in each administrative village jointly considering distribution and levels of economic development. 20–25 farmers were randomly selected for interview in each sample village. Taking into account the low education levels of the rural people in China, we conducted a face-to-face interview, with each interview lasting about one hour to ensure that every respondent could better understand the questions. In the end, we approached 700 households and managed to complete 688 questionnaires, for a 98.28% response rate.

The questionnaire was built on the theory of extended planned behavior (TPB), value-belief-norm (VBN), and utility analysis. All reflecting scales and indicators were adopted from previous studies. The contents of the questionnaire include socio-demographic background, and waste management behavior, consisting of a willingness to sort and pay, and frequency of waste separation; perceived benefit and perceived cost toward the WSB and policy instruments consisting of infrastructure, information campaign, and incentives policy. Of the respondents, 48.11% were male, and 51.89% were female. In terms of education level and age, the interviewed farmers were mainly over 40 years old (76.02%) with a low education level (80.67% with junior high school/technical school or below). The average age of the sample was 50.3 years, which was consistent with the current situation of rural China exhibiting a ‘village-hollowing’. It also shows that the main participants of rural domestic waste classification were aging farmers. The respondents with a yearly household income of 40,000 yuan and below accounted for 38.81% of the total sample, with an average annual income of 65,400 yuan (The average income of urban residents in Shaanxi Province was 95,511 yuan. Published by the Shaanxi Province Bureau of Statistics at http://tjj.shaanxi.gov.cn/). Details are shown in Table 1.

## 4. Methodology

### 4.1. Model Design

Based on the literature review and hypothesis, a model of perceived value effect on household waste classification was constructed. Because the dependent variable was household WSB measured by a 5-level scale, with a value of 1–5, the ordinary estimation method was not applicable. Therefore, we used an ordered logit model to test the impact of perceived benefit and perceived cost on waste classification behavior with maximum likelihood estimation [41]. The function is as follows.

(2) $$Y_{i}=\alpha_{0}+\alpha_{1}PB_{i}+\alpha_{2}PC_{i}+\alpha_{3}Control_{i}+\varepsilon_{i}$$

In the function, $Y_{i}$ is the household waste classification behavior. Specifically, $PB_{i}$ includes the spiritual and material benefits of the perceived benefits. $PC_{i}$ is the perceived cost in terms of time cost, energy, learning, and material. $Control_{i}\;$ includes 3 variables: Demographic characteristics of respondents, past waste disposal habit, and government support.$\;\varepsilon_{i}$ is the residual error, and $\;\alpha_{i}$ is the parameters to be estimated. This paper used STATA 16.0 (StataCorp LP., College Station, TX, USA) for ordered logit regression and IBM SPSS 24.0 (Chicago, IL, USA) for PCA.

### 4.2. Dependent Variable Measurement

To measure farmers’ WSB, this paper used the question item in the questionnaire—‘How often do you classify waste?’—based on a Likert scale. The respondents answered the frequency of waste sorting based on a range: Never sorting = 1, occasionally sorting = 2, neutral = 3, sometimes sorting = 4, always sorting = 5.

### 4.3. Independent Variable Measurement

#### 4.3.1. Perceived Benefit

Perceived benefit consists of spiritual benefit and material benefit [42,43]. The spiritual benefit was measured by 4 observable variables: Egoistic benefit, social-altruistic benefit, biospheric benefit, and reputation benefit. Biospheric benefit, also known as an environmental benefit, refers to the individual’s attention to the whole ecosystem. It was the estimated benefit of the behavior effect on the ecosystem [44,45]. Egoistic benefit and social-altruism represent a person’s concern for the well-being of himself and others, respectively [44,46]. To gain a reputation with one’s peers, an individual will adopt a ‘pro-social’ behavior of waste separation [7]. Hence, the degree of recognition by peers was used to measure reputation benefit [7]. The material benefit was measured by the monetary income that farmers could obtain in subsidy after waste classification or the incentive rewards in the form of lottery tickets and small gifts [47].

#### 4.3.2. Perceived Cost

Perceived cost includes material cost and non-material cost in terms of time cost, learning cost, and physical cost [16]. The time cost was measured by whether the waste classification process occupied the farmers’ time [36]. Waste classification was a relatively complex disposal process. Farmers with a low education level had to learn relevant knowledge and develop waste disposal skills to correctly sort the waste, which was defined as a study cost. When farmers performed waste classification, they not only separated the waste at home but also needed to deliver the recyclable garbage or valuable waste to the centralized recycling station. We defined these as physical costs. Waste sorting needs to be equipped with garbage bins, organic fertilizer, recycling equipment, or other infrastructure, which will increase the farmers’ perceived cost because of worrying about cost-sharing [48]; we define this as material cost. The details of the indicators are shown in Table 2.

All perceived benefit and perceived cost indicators are measured by a five-level scale (completely disagree = 1, quite disagree = 2, neutral = 3, quite agree = 4, completely agree = 5). The details of the indicators are shown in Table 2.

The analysis of reliability and validity of variables can reflect the quality and scientificity of the study. The Cronbach coefficient was used in reliability analysis. The Cronbach’s α values of the perceived benefit and perceives cost all exceeded 0.8, which exceeded the recommended value of 0.7 [49]. Convergent validity was used in the validity analysis. Specifically, convergent validity was tested by using the composite reliability (CR) and the average variance extracted (AVE). The results showed that the CR value ranged from 0.745 to 0.896, which exceeded an acceptable value (0.7) [50]. AVE was higher than the acceptable value of 0.5 [49], except PC. In addition, the values of factor loading of the perceived benefit and perceived cost exceeded 0.5 [50]; the values of KMO (Kaiser-Meyer-Olkin) exceed 0.6 [7]; Bartlett’s test was significant (*p* < 0.01). Generally speaking, the indicators of perceived value were highly reliable and had good validity. The details of indicators are shown in Table A2.

### 4.4. Control Variable Measurement

Studies have shown that gender, age, education level, and income level have a significant impact on waste classification behaviors [51,52]. Moreover, the past habits of household waste disposal will significantly affect waste classification behaviors [16,53]. Besides the factors of individual perspective, a policy instrument as a robust external factor could promote WSB. It includes providing waste classification facilities, economic incentives, and information campaigns [54,55,56]. When the government does not provide sufficient infrastructure such as garbage bins, the perceived inconvenience of individuals will hinder their willingness and behavior of waste classification [57,58]. The economic inducement policy, such as subsidies or gifts, can help to improve people’s enthusiasm for waste sorting [24,59]. The government distributes brochures and provides information to make participants realize their obligations in waste sorting and thus implement long-term waste classification [60,61]. Therefore, in this paper, the control variables affecting the waste classification behavior were summarized into 3 categories: Demographic characteristics of farmers include gender, age, education level, and income level. The past waste disposal habits were measured by asking farmers whether they had the habits of selling waste products to the recycling company. Policy instrument was divided into infrastructure, incentives policy, and information [13,62]. Infrastructure and information were measured by a 5-level scale, while incentive policy was measured by dichotomies value. The definitions and descriptive statistics of the variables are shown in Table 3.

According to the results of Table 3, the average value of household waste classification behaviors was 2.61, which means the frequency of household waste classification behavior was generally low. The descriptive statistics results of a policy instrument show that the local government provides much information to properly implement waste classification policy (the average value is 4.18) and provides quite sufficient waste classification infrastructure (the average value is 3.40). Comparing with infrastructure and information, the incentive policy for waste classification was lower (the average value is 0.28).

## 5. Results

The purpose of the paper is to investigate the impact of perceived benefit and perceived cost on waste classification behaviors. Before regression, the collinearity problem among variables should be tested. Hence, a collinearity test of variables was conducted. The estimated results showed that the variance inflation factor (VIF) values of all variables were less than 2 (details in Table A1 in Appendix A), indicating that the possibility of multi-collinearity among variables was very low [63]. Then, we tested the effects of perceived value on WSB by an ordered logit model. Table 4 summarizes the results of the ordered logit regression.

### 5.1. Influence of Perceived Benefit on WSB

Spiritual benefit has a significantly positive effect on waste classification behaviors at the level of 5%, which indicates that the higher the degree of spiritual benefits in terms of biospheric benefit, egoistic benefit, social-altruistic benefit, and reputation benefit, the higher the frequency of waste classification behaviors [64,65]. Based on the risk probability value, the frequency of waste classification of farmers with high spiritual income was 1.2 times that of lower ones. Hypothesis H1a was proven. Material benefit had no significant positive effect on waste classification behavior, and hypothesis H1b was not proven.

### 5.2. Influence of Perceived Cost on WSB

The time cost was negatively correlated with waste classification behaviors at the 1% level, which indicated that time cost was a barrier for farmers to participate in waste classification. The probability of farmers with higher perceived time cost to participate in waste classification was 0.78 times that of the lower group, and H2a was verified [17,36]. Learning cost had no significant effect on waste classification behaviors. In general, although the sample farmers were older and less educated, perceived learning cost did not constrain their participation in waste classification, and H2b was not proven. Physical cost significantly negatively impacted waste classification behaviors at the level of 10%. The probability of participating in waste sorting of farmers with higher perceived physical cost was 0.71 times that of the lower group. H2c was proven. The material cost had a significantly negative effect on waste classification behaviors at the level of 10%. Therefore, H2d was proven [60]. The risk probability value shows that the farmers with higher perceived material costs were 0.65 times that of lower farmers in waste classification frequency.

### 5.3. Influence of Control Variables on WSB

Gender and age had no significant effects on WSB [3]. Education level had a significantly negative correlation with WSB at the level of 10%, and the frequency of waste sorting of households with a higher education level was 0.66 times that of lower-level households. Annual household revenue had a negative effect on waste classification behaviors at the level of 5%. The risk probability value showed that the probability of classification behaviors of farmers with high-level income was 0.45 times that of farmers with low-level income. The past garbage disposal habit and the behavior of selling waste to recycling companies had a significantly positive correlation with the waste classification behaviors of farmers at the level of 1%, which means that farmers who had the habit of selling waste were more likely to participate in waste classification [16,53].

For policy instrument variables, the infrastructure significantly positively affected waste classification behaviors at the level of 1%. The risk probability coefficient showed that when the basic waste disposal facilities were sufficiently equipped, the probability of household WSB was 1.48 times that of insufficient infrastructure [57,58]. The incentive policy had a significantly positive effect on WSB at the level of 1%. Higher incentive policies increased the probability of WSB by 4.47 times that of lower ones [24,59]. Information had no significant impact on WSB, and this was inconsistent with the previous studies [64,65].

### 5.4. Investigating the Effect of Perceived Value on WSB at Different Income Levels

The maximization of personal utility was constrained by income. Therefore, to explore the influence path of perceived value on behaviors under different income levels, this paper takes the per capita income of Shaanxi Province published in the statistical yearbook of Shaanxi Province in 2018 as the benchmark (Published by the Shaanxi Province Bureau of Statistics at http://tjj.shaanxi.gov.cn/) and divided the farmers into two levels. Higher than the per capita of Shaanxi Province is defined as the high-income group, and lower ones are defined as the low-income group. We tried to discuss the different effects of perceived benefit and perceived cost on waste classification behavior at different income levels. The statistical results are shown in Table 5.

The results of testing group differences of income level show that spiritual benefit had a significantly positive impact on the WSB of households in the low-income group. However, it had no significant impact on the high-income group, which indicates that the influence pathway of spiritual benefit on WSB in high-and low-income groups was different. Physical cost had a significantly negative correlation with WSB in the low-income group and the high-income group at the level of 10% and 5%, respectively. The impact of time cost on the WSB in the low-income group was not significant, but the negative impact on the high-income group was significant at the level of 1%. The risk probability value shows that the WSB of farmers with highly perceived time cost was 0.35 times than that of lower ones. Learning cost had a significantly negative impact on WSB in the low-income group at the level of 5%, but it had not passed the significance test for the high-income group. The material cost had a significantly positive impact on farmers’ WSB in the low-income group at the level of 1%, while it had a significantly negative impact on WSB in the high-income farmers at the level of 5%.

## 6. Discussion

The purpose of this paper was to analyze the effects of perceived benefit and perceived cost of perceived value on household WSB. Measurement scales of perceived benefit and perceived cost were developed, ordered logit regression, and cluster regression was introduced. The results confirmed that the perceived benefit played a positive role, and the perceived cost played a negative role in the WSB. This is consistent with the studies by Li et al. [31] and Lee et al. [36]. Furthermore, perceived benefits and perceived cost have different effects on WSB in high- and low-income groups.

From the perspective of perceived benefit, the spiritual benefit has a positive effect on the WSB. Material benefit has no significant effect on the WSB. This is inconsistent with the previous study [52]. The possible reasons are as follows. Firstly, the material benefit from selling recyclable garbage and disposing of decaying garbage is small; therefore, it could not reach the threshold value of encouraging households to sort waste. Secondly, the incentive policies provided by the government, such as gifts or subsidies, are too low to make farmers perceive the material benefits.

From the perspective of perceived cost, time cost, physical cost and material cost have negative effects on the WSB, and learning cost has no significant effects on the WSB. For time cost, the farmers spend much time in heavy agricultural production in rural china, and hence, when they consider that waste sorting is a waste of time, they tend to refuse waste sorting. For the physical cost, combined with descriptive statistics and the current situation of ‘hollowing out’ in rural areas, the main participants of waste treatment are generally older farmers. Comparing with energetic young people, older people will perceive a higher physical cost during the complex classification process—it will hinder their participation in waste sorting. The possible reasons for material costs are as follows. Firstly, due to the unbalanced supply of public goods and services, the level of policy instruments varies greatly in different rural areas, and the situation of insufficient classification facilities and low frequency of centralized recycling still exists [66]. Consequently, farmers have to buy classified garbage cans or establish temporary garbage dumps, which leads to additional expenses and increases households’ material costs. Secondly, because households predict a cost-sharing of waste classification, they will worry that the implementation of waste classification may lead to an extra waste treatment fee and the cost-sharing of recyclable facilities provided by the government. Hence, the increase in perceived material cost will reduce farmers’ enthusiasm to participate in waste classification.

From the perspective of the heterogeneity effect of income, spiritual benefit, physical cost, learning cost, and material cost are the main factors of perceived value affecting the WSB in the low-income group. Time cost, physical cost, material cost are the main factors of perceived value affecting the WSB in the high -income group. A possible reason is that the higher the material cost of households in the low-income group for waste classification, the stronger the willingness to continue to participate in waste classification, thus showing the higher classification frequency because of loss aversion. However, households with higher perceived material cost have a lower frequency of participating in waste classification when they think waste sorting is troublesome or time-consuming. In summary, time cost, learning cost, and material cost show different influence effects on WSB in high- and low-income groups.

Besides, for the control variable, education level has a significantly negative correlation with WSB, inconsistent with previous studies [51,55]. The possible reason is that the implementation of the waste classification policy in pilot areas is compulsory, and farmers with a lower education level may more easily subordinate to the policy than more educated ones. Annual household income has a negative effect on WSB. The possible reason is that the farmers at a lower income level are more likely to be motivated by material benefit and thus more actively participate in waste classification than those at a higher income level. Information on policy instruments has no significant impact on WSB. Some research shows that the longer the promotion time of social publicity activities, the higher the level of waste classification of individuals [4]. Therefore, the possible reason is that the pilot areas are in the early stage of waste sorting because waste sorting policy was implemented in 2017, and the publicity and promotion time is short, thus the impact of information on waste classification behaviors is not fully realized.

## 7. Conclusion and Policy Implications

### 7.1. Conclusions

Based on the utility maximization theory of the rational economic man, this paper analyses the effect mechanism of perceived value on household waste classification behavior from the perspective of benefit and cost analysis. Then, it investigates the group differences of perceived value on WSB of households in high- and low-income groups by using 688 household survey data from three cities and three counties in the Shaanxi Province. The conclusions are as follows.

Firstly, the perceived value has a significant impact on the household waste classification behavior. Specifically, the perceived benefit has a significantly positive impact on household waste classification behavior, and the perceived cost has a significantly negative impact on WSB. The spiritual benefit of the perceived benefit has a significantly positive impact on the household waste classification behavior, while the material income of the perceived benefit has no significant positive impact on the WSB. Time cost, physical cost, and material cost of the perceived cost have a significantly negative impact on household waste classification behaviors, while learning cost has not passed the significance test.

Secondly, the impact of perceived value on waste classification behavior of households at different income levels is different. Specifically, the spiritual benefit has a significantly positive impact on WSB of households in the low-income group, but it has no significant impact on WSB in the higher income group. The learning cost, physical cost, and material cost of the perceived cost are the important factors influencing the waste classification behavior in the low-income group, while the time cost, physical cost, and material cost affect the farmers’ participation in waste separation in the high-income group.

Thirdly, besides the perceived benefit and perceived cost, gender and age have no significant effects on WSB. Education levels had a significantly negative correlation with WSB. The past garbage disposal habit of households has a significantly positive correlation with the waste classification behaviors. For policy instrument variables, the infrastructure and incentive policy significantly positively affect waste classification behaviors. However, the information has no significant impact on the WSB.

### 7.2. Policy Implications

This study has some policy implications. The government should enhance the enthusiasm of rural residents to participate in domestic waste classification by exploring the dual perspective of perceived benefit and perceived cost. The countermeasures are as follows.

Firstly, the government could provide information in terms of the importance, methods, and welfare effects of waste classification, combining ‘offline’ and ‘online’ modes, which could enhance the spiritual benefits of farmers and reduce their learning costs. Specifically, from the perspective of an offline mode, the local government could establish a classified guide policy, which could provide face-to-face publicity, guidance, inspection, and supervision with the help of classified guiders. From the perspective of an online mode, the government could encourage technical persons or companies to develop a cell phone application of waste classification to provide relevant waste sorting knowledge flexibly and vividly.

Secondly, the local government could establish a new management model, the supervision of the block head. Specifically, considering the geographical location of the areas, the village government should be divided into several blocks and select a farmer with a good reputation as a block head for each block. The block head is responsible for supervising the situation of waste classification in the region, and the block head should inspect, inform the situation of waste classification in time, and make the corresponding encouragement and punishment decisions according to the inspection results, which could improve the perceived benefit in the perspective of reputation benefit.

Thirdly, for the purposes of improving the perceived material benefit of households, the government can expand the recycling pathways of perishable waste and recyclable waste through third-party recycling companies or by public-private partnership projects to increase the waste recycling rate. The current situation of rural waste treatment is that a large quantity of recyclable and perishable waste available for sale to recycling companies is not collectible due to the lack of recycling channels. In the field investigation, we found that only the plastic film used to cover the plastic greenhouse has a stable recycling channel among the numerous recyclable wastes. Hence, it is necessary to expand the recycling pathways to increase the recycling rate for waste reduction and to improve the perceived material benefit to encourage more households to participate in waste sorting.

Finally, considering the different impacts of perceived value on WSB at different income levels, it is suggested that the relevant departments should flexibly set up a variety of classification modes according to the economic development status of rural areas, the income level of farmers, and the acceptance degree and cooperation of farmers for waste classification. Specifically, the two-categories sorting mode of recyclable and non-recyclable or the three-categories sorting mode of decomposed waste, toxic waste, and other waste can be adopted in areas with a high income level. However, a four-categories sorting mode can be adopted in low-level income areas, or even a six-categories sorting mode or more. Meanwhile, the government should take more responsibility for the allocation of garbage sorting facilities to reduce the material cost perception of farmers in the areas with a low economic development level.

## Figures and Tables

**Table 1:** Demographic characteristics of samples (N = 688).

Demographics	Categories	Frequency	Percent
Gender	Male	331	48.11
	Female	357	51.89
Age	18–30	66	9.59
	31–39	99	14.39
	40–49	174	25.29
	50–59	187	27.18
	60 and above	162	23.55
Education	Illiterate	78	11.34
	Primary education	186	27.03
	Junior middle school/technical school	291	42.30
	High school/secondary specialized school	111	16.13
	Graduates and above	22	3.20
Income (annual in RMB)	Under 40,000	267	38.81
	40,001–80,000	241	35.03
	80,001–120,000	101	14.68
	More than 120,000	79	11.48

**Table 2:** Definition and descriptive statistics of dependent variables (N = 688).

Variables	Items	Measure Items	Mean	SD
Perceived benefit	Spiritual benefit	Egoistic benefit: Solid waste separation is good for you (SB1)	4.621	0.680
		Social-altruistic benefit: Solid waste separation is good for others (SB2)	4.562	0.677
		Biospheric benefit: Solid waste separation is good for the ecological environment (SB3)	4.523	0.697
		Reputation benefit: Solid waste separation is helpful to improve your reputation in the village (SB4)	4.492	0.712
	Material benefit	A gift or subsidies from the government for garbage sorting can motivate you to do sorting (MB1)	4.511	0.757
		The benefits of selling recyclable waste can motivate you to do sorting (MB2)	4.372	0.758
		Selling perishable or economically valuable waste can motivate you to do sorting (MB3)	4.341	0.760
Perceived cost	Time cost	Solid waste separation wastes too much time (TC1)	2.201	1.318
		Delivering perishable waste to composting sites wastes too much time (TC2)	2.217	1.322
		Delivering classified waste to a centralized collection point wastes too much time (TC3)	2.745	1.416
	Learning cost	It is difficult for you to master the method of waste sorting (LC1)	4.012	1.187
		It is difficult for you to master the composting technology of decaying waste (LC2)	4.001	1.216
		It is difficult for you to use an intelligent garbage collection device (LC3)	3.503	1.431
	Physical cost	Solid waste separation wastes too much energy (PC1)	4.001	1.279
		Delivering the perishable waste to composting sites wastes too much energy (PC2)	3.971	1.245
		Delivering classified waste to a centralized collection point wastes too much energy (PC3)	3.162	1.606
	Material cost	Building up a temporary dump costs you an extra expenditure (MC1)	2.211	1.315
		Waste sorting causes your garbage charges to increase (MC2)	2.772	1.395
		Preparing a sorting bin will cost you an extra expenditure (MC3)	2.793	1.384

**Table 3:** Definition and descriptive statistics of control variables.

Variables		Items	Definition	Mean	SD
Dependent variable	WSB		How often do you classify waste? Never sorting = 1, occasionally sorting = 2, neutral = 3, sometimes sorting = 4, always sorting = 5	2.612	1.500
Independent variable	Perceived benefit	Spiritual benefit	Factor score	0.000	1.000
		Material benefit	Factor score	0.000	1.000
	Perceived cost	Time cost	Factor score	0.000	1.000
		Learning cost	Factor score	0.000	1.000
		Physical cost	Factor score	0.000	1.000
		Material cost	Factor score	0.000	1.000
Control variables	Demographic characteristics	Gender	Female = 0, male = 1	0.480	0.500
		Age	The age of the respondent	50.330	12.694
		Education level	Illiterate = 1, primary education = 2, junior middle school/technical school = 3, high school/secondary specialised school = 4, graduates and above = 5	1.760	1.019
		Income level (annual in RMB)	Annual income of the household (10,000/RMB)	6.541	7.635
	Past waste disposal habitshabi		How often have you ever sold waste products in the past? (never = 1, occasionally = 2, neutral = 3, often = 4, always = 5)	3.360	0.721
	Government support	Infrastructure	The village committee provides sufficient waste facilities (including separation bins, centralised recycling point, and so on) (completely disagree = 1, quite disagree = 2, neutral = 3, quite agree = 4, completely agree = 5)	3.397	1.267
		Incentives policy	Does your village provide material incentives for waste classification? (including small gift or cash rewards) (no = 0, yes = 1)	0.281	0.452
		Information	Does your village carry out information campaigns on waste sorting (never = 1, occasionally = 2, neutral = 3, often = 4, always = 5)	4.182	0.954

**Table 4:** The statistical value of influencing factors on the household waste sorting behavior.

Variables	Coefficient	Standard Error	Risk Ratio	Standard Error
Spiritual benefit	0.198 **	0.076	1.219 **	0.093
Material benefit	0.055	0.075	1.056	0.080
Time cost	−0.253 ***	0.079	0.777 ***	0.061
Learning cost	−0.107	0.072	0.899	0.065
Physical cost	−0.345 *	0.192	0.709 *	0.136
Material cost	−0.426 *	0.223	0.653 *	0.145
Gender	−0.140	0.151	0.869	0.131
Age	0.001	0.006	1.001	0.006
Education level	−0.416 *	0.252	0.660 *	0.166
Income level (annual in RMB)	−0.794 **	0.389	0.452 **	0.176
Past waste disposal habits	0.431 ***	0.107	1.540 ***	0.165
Infrastructure	0.393 ***	0.067	1.481 ***	0.098
Incentives policy	1.498 ***	0.230	4.472 ***	1.028
Information	−0.121	0.074	0.886	0.066
cut1	−7.897		4.413	
cut2	−6.619		4.413	
cut3	−6.082		4.416	
cut4	−4.856		4.425	
N	688			
Pseudo R^2^	0.121			

Note: *, **, and *** donate a statistical significance at the 10%, 5%, 1%, respectively.

**Table 5:** Effects of perceived benefit and perceived cost of waste sorting behavior at different income levels.

	Low-Income Group		High-Income Group	
	Coefficient	Risk Ratio	Coefficient	Risk Ratio
Spiritual benefit	0.298 **	1.347 **	0.106	1.111
	(0.112)	(0.146)	(0.119)	(0.128)
Material benefit	0.104	1.109	0.0190	1.019
	(0.112)	(0.121)	(0.107)	(0.110)
Time cost	−0.170	0.844	−1.035 ***	0.355 ***
	(0.123)	(0.093)	(0.369)	(0.108)
Learning cost	−0.313 **	0.731 **	−0.103	0.902
	(0.111)	(0.079)	(0.107)	(0.096)
Physical cost	−2.559 *	0.077 *	−0.476 **	0.622 **
	(1.199)	(0.106)	(0.297)	(0.139)
Material cost	2.351 ***	10.499 ***	−0.297 **	0.743 **
	(0.635)	(6.762)	(0.109)	(0.081)
Control variables	Controlled		Controlled	
cut1	72.030		−22.375	
	(23.252)		(5.054)	
cut2	73.345		−20.965	
	(23.266)		(5.041)	
cut3	73.855		−20.314	
	(23.284)		(5.035)	
cut4	75.328		−19.164	
	(23.325)		(5.020)	
N	341		347	
R2	0.119		0.166	

Note: *, **, and *** donate a statistical significance at the 10%, 5%, 1%, respectively.

## Appendix A

**Table A1:** Multi-collinearity test.

Items	Tolerance	VIF	Items	Tolerance	VIF
Spiritual benefit	0.653	1.532	Age	0.855	1.170
Material benefit	0.645	1.550	Education level	0.816	1.225
Time cost	0.914	1.094	Income level	0.914	1.094
Learning cost	0.754	1.326	Past waste disposal habits	0.812	1.231
Physical cost	0.797	1.254	Infrastructure	0.838	1.193
Material cost	0.984	1.016	Incentives policy	0.746	1.340
Gender	0.942	1.062	Information	0.944	1.059

**Table A2:** Results of factor analysis.

Variables	Items	Measure Items	Factor Loading	KMO	Bartlett’s TEST	Cronbach’s α	Average Variance Extracted	CR
Perceived benefit	Spiritual benefit	SB1	0.650	0.824	1485.000 (0.000)	0.881	0.548	0.828
		SB2	0.760					
		SB3	0.799					
		SB4	0.744					
	Material benefit	MB1	0.839	0.738	1131.659 (0.000)	0.883	0.657	0.852
		MB2	0.813					
		MB3	0.778					
Perceived cost	Time cost	TC1	0.909	0.653	2056.627 (0.000)	0.892	0.705	0.875
		TC2	0.925					
		TC3	0.658					
	Learning cost	LC1	0.847	0.623	1088.289 (0.000)	0.810	0.574	0.794
		LC2	0.862					
		LC3	0.510					
	Physical cost	PC1	0.711	0.669	643.791 (0.000)	0.893	0.498	0.745
		PC2	0.785					
		PC3	0.611					
	Material cost	MC1	0.659	0.647	1839.487 (0.000)	0.893	0.694	0.896
		MC2	0.923					
		MC3	0.892					

**Questionnaire**

**Table A3:** Perceived value in the questionnaire. (Completely disagree = 1, quite disagree = 2, neutral = 3, quite agree = 4, completely agree = 5).

Variables	Definition	Options				
		1	2	3	4	5
Spiritual benefit	Egoistic benefit: Solid waste separation is good for you (SB1)					
	Social-altruistic benefit: Solid waste separation is good for others (SB2)					
	Biospheric benefit: Solid waste separation is good for the ecological environment (SB3)					
	Reputation benefit: Solid waste separation is helpful to improve your reputation in the village (SB4)					
Material benefit	A gift or subsidies from the government for garbage sorting can motivate you to do sorting (MB1)					
	The benefits of selling recyclable waste can motivate you to do sorting (MB2)					
	Selling perishable or economically valuable waste can motivate you to do sorting (MB3)					
Time cost	Solid waste separation wastes too much time (TC1)					
	Delivering perishable waste to composting sites wastes too much time (TC2)					
	Delivering classified waste to a centralized collection point wastes too much time (TC3)					
Learning cost	It is difficult for you to master the method of waste sorting (LC1)					
	It is difficult for you to master the composting technology of decaying waste (LC2)					
	It is difficult for you to use an intelligent garbage collection device (LC3)					
Physical cost	Solid waste separation wastes too much energy (PC1)					
	Delivering the perishable waste to composting sites wastes too much energy (PC2)					
	Delivering classified waste to a centralized collection point wastes too much energy (PC3)					
Material cost	Building up a temporary dump costs you an extra expenditure (MC1)					
	Waste sorting causes your garbage charges to increase (MC2)					
	Preparing a sorting bin will cost you an extra expenditure (MC3)					
